# A Network Pharmacology Approach to Understanding the Mechanisms of Action of Traditional Medicine: Bushenhuoxue Formula for Treatment of Chronic Kidney Disease

**DOI:** 10.1371/journal.pone.0089123

**Published:** 2014-03-05

**Authors:** Shao-hua Shi, Yue-piao Cai, Xiao-jun Cai, Xiao-yong Zheng, Dong-sheng Cao, Fa-qing Ye, Zheng Xiang

**Affiliations:** 1 School of Pharmaceutical Sciences, Wenzhou Medical University, Wenzhou, China; 2 School of Pharmaceutical Sciences, Central South University, Changsha, China; Universidade Federal do Rio de Janeiro, Brazil

## Abstract

Traditional Chinese medicine (TCM) has unique therapeutic effects for complex chronic diseases. However, for the lack of an effective systematic approach, the research progress on the effective substances and pharmacological mechanism of action has been very slow. In this paper, by incorporating network biology, bioinformatics and chemoinformatics methods, an integrated approach was proposed to systematically investigate and explain the pharmacological mechanism of action and effective substances of TCM. This approach includes the following main steps: First, based on the known drug targets, network biology was used to screen out putative drug targets; Second, the molecular docking method was used to calculate whether the molecules from TCM and drug targets related to chronic kidney diseases (CKD) interact or not; Third, according to the result of molecular docking, natural product-target network, main component-target network and compound-target network were constructed; Finally, through analysis of network characteristics and literature mining, potential effective multi-components and their synergistic mechanism were putatively identified and uncovered. Bu-shen-Huo-xue formula (BSHX) which was frequently used for treating CKD, was used as the case to demonstrate reliability of our proposed approach. The results show that BSHX has the therapeutic effect by using multi-channel network regulation, such as regulating the coagulation and fibrinolytic balance, and the expression of inflammatory factors, inhibiting abnormal ECM accumulation. Tanshinone IIA, rhein, curcumin, calycosin and quercetin may be potential effective ingredients of BSHX. This research shows that the integration approach can be an effective means for discovering active substances and revealing their pharmacological mechanisms of TCM.

## Introduction

Chronic complex diseases such as cancer, diabetes, cardiovascular diseases and neuropsychiatric diseases are a great threat to human health and survival. They have become one of the most important social problems in the world. Traditional Chinese medicine (TCM) has been used as the main therapy means of diseases in China for thousands of years. Through the synergistic effect of multi-components, multi-channels and multi-targets, it showed significant advantages over a single drug treatments, especially for the treatment of chronic complex, multi-factorial diseases [1], [2]. Compared to western medicine, it has been observed to have lower side effects [3]–[6]. However, for a long time, due to complex chemical composition, prescription diversity and the lack of an effective research approach, the investigation of active compounds and pharmacological mechanisms of action has encountered great challenges, thus becoming one of the main bottlenecks of modernization and internationalization of TCM.

The discovery methods of active compounds from TCM are primarily the separation and extraction of different ingredients in TCM, and then to test the biological activity of each compound, and finally to elucidate its pharmacological mechanism of action. However, due to numerous chemical substances from TCM, the separation and screening for each ingredient was time-consuming and costly. Furthermore, TCM is a multi-component synergistic system and plays a therapeutic role through the overall regulation on diseases. Therefore, the traditional approach of drug discovery has many limitations to the study of multi-component combination drugs and their pharmacological mechanism of action [7].

In recent 10 years, chemoinformatics methods are successfully used to study TCM. Wong *et al* have proposed a stochastic search algorithm to define the most promising combinations from a large number of possibilities [2]. Chen *et al* constructed an artificial neural network based quantitative composition-activity relationship (QCAR) model in order to design and optimize the proportion of two active components from Qi-Xue-Bing-Zhi-Fang, evidently the optimal combination exhibited increased efficiency over the original formula [8]. These studies indicates that chemoinformatics methods have the ability to deal with some problems related to multi-component drug discovery.

With the development of systems biology, network biology and polypharmacology, Andrew L Hopkins put forward the concept of network pharmacology [9]. From the viewpoint of network level, network pharmacology aims at investigating drug to disease intervention or influence, and reveals the synergism law of multi-component drugs, in order to find the high efficiency and low toxicity of multi-target drugs. Therefore, from the molecular level, the idea of TCM was consistent with those of network pharmacology. Presently, many medical experts pay more attention to network pharmacology and it has been successfully used in the discovery of effective components and the pharmacological mechanism of action in TCM. Xu *et al* employed a molecular docking protocol and network analysis to study the interaction of natural compounds from Tongguan Capsule that treated coronary heart disease, and was finally able to screen for the potentially effective components [10]. Ye’s group and Zhang’s group employed inverse molecular docking program and network modeling methods to identify direct interacting targets of salvianolic acid B [11] and astragaloside IV [12], respectively. Li’s group established an algorithm called NIMS (Network target based Identification of Multi-component Synergy) to prioritize synergistic agent combinations and then characterized the potential mechanisms of multi-component synergy from a network target perspective [13]. Wang *et al* employed molecular docking and bioinformatics methods to study Danshen Formula on the effect of treatment for cardiovascular disease, which made a contribution to a better understanding of its mechanisms [14]. Both network and cheminformatics approaches are powerful tools for finding and elucidating active multi-components and their mechanisms of action. They provide new ways for the research on multi-component activity and pharmacological mechanism of action.

Disease networks constructed by network biology methods were powerful tools for screening out candidate drug targets [15], [16]. Bioinformatics can mine and comment on the biological information of disease network. Molecular docking method can determine the binding level between molecules and target proteins. The combination of these three methods can contribute to the comprehensive understanding of the characteristics and law of TCM from global network viewpoints. At the same time, it provides even more knowledge for fully revealing the active compounds and their mechanisms of action.

An estimated 10% of the adult population in the world has some degree of chronic kidney disease (CKD) [17], [18] and a considerable proportion of cases eventually progress to end-stage kidney failure, which requires lifelong dialysis or kidney transplantation. As a result, CKD has become a major public health problem transpiring on a global scale, which caused enormous socioeconomic burdens on the affected individuals, families and societies. BSHX, designed on the basic principles of TCM and comprised of five herbs capable of treating chronic kidney disease, including *Astragali radix* (AR) [19]–[21], *Curcumae rhizoma* (CR) [22]–[24], *Rhei radix et rhizoma* (RR) [25]–[28], *Cuscutae semen* (CS) [29], and *Salviae miltiorrhizae radix et rhizoma* (SM) [30]–[33], is a clinical medicine frequently used for the treatment of CKD because of its lower risk of side effects when compared with chemical drugs, such as enalapril, amlodipine, and metoprolol [3], [34], [35]. However, the effective compounds and their pharmacological mechanism of action remained unclear. In this paper, an integrated approach of network biology, functional gene pathway analysis, network analysis and molecular docking method, were used to reveal candidate drug targets related to CKD, active compounds from BSHX and their pharmacological mechanisms of action.

## Materials and Methods

**Figure 1:**
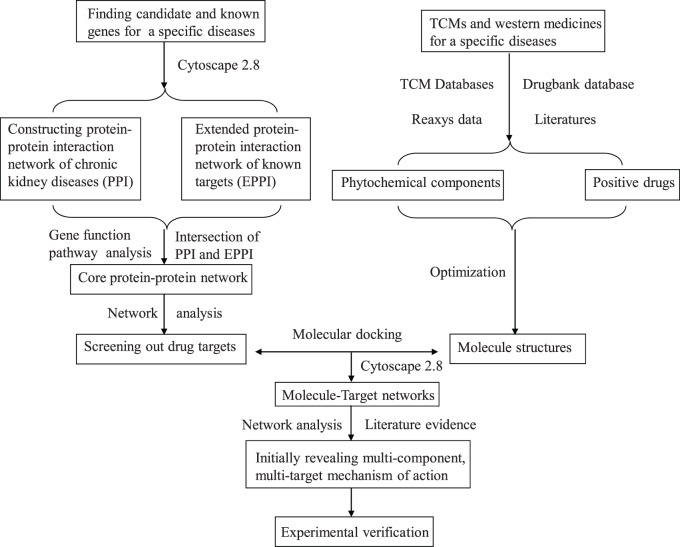
The whole framework based on an integration strategy of network pharmacology.

Our protocol involved five main steps: (1) finding known targets and candidate genes related to CKD; (2) finding phytochemical ingredients of BSHX from the literature database and public repository; (3) performing the molecular docking and constructing a natural product-target network; (4) constructing protein-protein networks (PPIs) and elucidating a biological function analysis; (5) constructing different types of molecule-target networks and analyzing these networks. (6) Some validation of data regarding disease treatment. The whole framework is shown in Fig. 1.

### Finding Candidate Genes and Known Drug Targets

Genes associated with CKD were collected by searching the Online Mendelian Inheritance in Man (OMIM) [36] database with keyword ‘renal or kidney’, Genetic Association Database (GAD) [37] with keyword ‘chronic kidney disease’ and from the differentially expressed genes of microarray experiments of Gene Expression Omnibus (GEO) database [38]. In the OMIM database, the searched genes unrelated to CKD, such as renal carcinoma gene etc, were manually deleted. In the GEO database, microarray experiments related to CKD were used: GEO series (GSE) 7392 and 22459. The GSE7392 experiment was a comparison of molecular evidence of injury and inflammation in normal and fibrotic renal allografts one year post-transplant, and the GSE22459 experiments are investigations of fibrosis with inflammation at one year from transplant functional decline. The algorithm of significance analysis of microarrays [39] in array-tool (version 3.7) was used to extract the significant genes associated with CKD from the microarray experiments. We selected genes for which the false discovery rate was less than 0.01. The list of all selected genes was provided on the supplementary materials (Table S1). In addition, 31 therapeutic target proteins associated with CKD, used as known target proteins (Table 1), were collected from Therapeutic Target database [40] and DrugBank [41]. All used dataset were downloaded on November 9, 2011.

**Table 1:** The information of the known and putative targets, positive drugs and cuttoff values.

Protein	Targets Name	Gene Symbol	PDB-ID	Positive Drugs		Cutoff Value	No^a^
Known target proteins	Carbonic anhydrase II	CA2	1BN3	Topiramate		−8.4	10
	Raf kinase	RAF1	1C1Y	Sorafenib		−9.6	12
	Macrophage migration inhibitory factor	MIF	1GCZ	ethyl 7-hydroxy-2-oxochromene-3-carboxylate		−8.9	10
	Hepatocyte growth factor	HGF	1GMO	N,O6-Disulfo-Glucosamine		−7	79
	Hypoxia-inducible factor 1α	HIF1A	1H2K	Everolimus		−6.3	66
	Soluble epoxide hydrolase	EPHX2	1ZD3	4-{[(cyclohexylamino)carbonyl]amino} butanoic acid		−7.4	15
	Carbonic anhydrase XII	CA12	1JD0	Hydrochlorothiazide		−6.9	26
	Peroxisome proliferator-activated receptor	PPARG	1K74	Fenofibrate		−9.4	39
	Angiotensin converting enzyme	ACE	1O86	Candoxatril		−9	74
	Monoamine oxidase B	MAOB	1OJ9	Pargyline		−9.2	39
	Mitogen-activated protein kinase 1	MAPK1	1PME	4-[4-(4-fluorophenyl)-2-[4-[(S)-methylsulfinyl]phenyl]-1H-imidazol-5-yl] pyridine		−9.4	27
	Mast stem cell growth factor receptor	KIT	1T46	Sorafenib		−10.1	34
	Thymidine phosphorylase	TYMP	1UOU	Chloro-6-[(2-Iminopyrrolidin-1-Yl)Methyl]Pyrimidine-2,4(1 h,3 h)-Dione		−7.7	76
	Macrophage metalloelastase	MMP12	1UTT	(6R)-4-benzyl-6-(1-methyl-2,2-dioxido-1,3-dihydro-2,1-benzisothiazol-5-yl)morpholin-3-one		−10.6	6
	β2 adrenergic receptor	ADRB2	3D4S	Carvedilol		−9.3	45
	Adenosine A2α receptor	ADORA2A	3EML	Mefloquine		−9.8	71
	C-C chemokine receptor type 1	CCR1	1Y5D	Maraviroc		−8.2	35
	Mitogen-activated protein kinase 14	MAPK14	1ZZ2	N-[(3z)-5-Tert-Butyl-2-Phenyl-1,2-Dihydro-3h-Pyrazol-3-Ylidene]-N**′**-(4-Chlorophenyl)Urea		−8.4	14
	Lymphocyte function-associated antigen	ITGAL	1CQP	Lovastatin		−7.2	0
	Vasopressin V1α receptor	AVPR1A	1YTV	Conivaptan		−10.5	20
	Placeta growth factor	PGF	1FZV	Suplatast tosylate		−5	25
	Transforming growth factor beta 1	TGFB1	1KLD	NO ligand			
	Tumor necrosis factor ligand superfamily member 5	TNFSF5	1I9R	NO ligand			
	Nuclear factor NF-κB	NFKB1	1NFI	NO ligand			
	Carbonic anhydrase IV	CA4	1ZNC	NO ligand			
	C-C motif chemokine-2	CCL2/MCP1	2BDN	NO ligand			
	DNA-directed RNA polymerase II 19 kDa polypeptide	POLR2D	2C35	NO ligand			
	Carbonic anhydrase IX	CA9	2HKF	NO ligand			
Known target proteins	Plasminogen activator inhibitor-1	SERPINE1	1OC0	NO ligand			
	RAC-α serine/threonine kinase	No symbol	1AO2	NO ligand			
	Protein-glutamine γ-glutamyltransferase	TGM2	2Q3Z	NO ligand			
Putative targets	Tyrosine-protein kinase BTK	BTK	3OCS	Staurosporine		−9.6	53
	Small inducible cytokine A5	CCL5	1U4M	Heparin_Disaccharide_I-S		−6	15
	Epidermal growth factor receptor	EGFR	2GS7	Flavopiridol		−9	19
	Estrogen receptor	ESR1	3Q97	Estradiol		−10	1
	Heat shock cognate 71 kDa protein	HSPA8	3FZK	(2R,3R,4S,5R)-2-[6-amino-8-[(3,4-dichlorophenyl)methylamino]purin-9-yl]-5-(hydroxymethyl)oxolane-3,4-diol		−8.2	34
	Insulin receptor	INSR	2HR7	Hydrochloride)		−9.5	21
	Proto-oncogene tyrosine-protein kinase LCK	LCK	3AC1	N-(2-chloro-6-methylphenyl)-8-[(3S)-3-methylpiperazin-1-yl]imidazo[1,5-a]quinoxalin-4-amine		−8.2	60
	Hepatocyte nuclear factor 4-alpha	HNF4A	1PZL	1_methyl_2-nitro_benzo[e]benzofuran		−7.4	79
	Glucocorticoid receptor	NR3C1	3K22	Flunisolide		−10	26
	Phosphatidylinositol-4,5-bisphosphate 3-kinase catalytic subunit alpha isoform	PIK3CA	3HHM	Wortmannin		−9.1	20
	Plasminogen activator, tissue	PLAT	1A5H	Iloprost		−8.5	87
	Acyl-CoA dehydrogenase family member 8, mitochondrial	ACTN1	1RX0	Methacrylyl-Coenzyme_A		−9	16
	Protein tyrosine phosphatase	PTPN1	1BZH	(Oxalyl-Amino)-Naphthalene-2-Carboxylic_Acid		−8	19
	Protein kinase C, beta	PRKCB	2I0E	Vitamin_E		−9.7	13
	E3 ubiquitin protein ligase	VHL	3ZRC	4-((naphthalen-2-ylamino)methyl)benzene-1,2-diol		−7.7	21
	FYN oncogene related to SRC, FGR, YES	FYN	1AOT	2,5,8,11-Tetraoxadodecane		−6.6	29
	9-mer from C-C chemokine receptor type 5	CCR5	2RLL	NO ligand			
	Fc fragment of IgG, low affinity IIb, receptor (CD32)	FCGR2B	2FCB	NO ligand			
	Fibronectin 1	FN1	3MQL	NO ligand			
	Myeloma immunoglobulin D lambda	IGHG1	1ZVO	NO ligand			
	Solute carrier family 4	SLC4A1	1BTT	NO ligand			
	Signal transducer and activator of transcription	STAT1	1BF5	NO ligand			
	Jun proto-oncogene	JUN	1FOS	NO ligand			
	KIAA0101	KIAA0101	No PDB data				
	Catechol-O-methyltransferase	COMT	No PDB data				
Protein	Targets Name	Gene Symbol	PDB-ID	Positive Drugs	Cutoff Value		No
Putative targets	Decorin	DCN	No PDB data				
	Clusterin	CLU	No PDB data				
	Transforming growth factor, beta receptor 1	TGFBR1	No PDB data				
	Interleukin 8	IL8	No PDB data				
	Apolipoprotein A–I	APOA1	No PDB data				
	Signal transducer and activator of transcription 5B	STAT5B	No PDB data				

No^a^: The number of effective molecular docking.

### Finding Phytochemical Components

The natural product data sets of AR, CR, RR, CS and SM, were collected from Comprehensive Natural Products in TCM [42] and Reaxys data (https://www.reaxys.com). These natural products were filtered by Lipinski’s ‘rule of five’ [43]. Drugs or molecule-protein complexes which were referred to as CKD targets, were used as positive drugs and were collected from Drugbank and Protein Data Bank [44], [45]. And then molecular docking scores of active ligands which were from drugs or molecular-protein complexes were defined as the cutoff value to screen out potential active ingredients from the natural product data set.

### Molecular Docking and Construction of Natural Product-target Network

All molecular structures were optimized by molecular mechanics optimization method based on MMFF94 force field, and the stop condition was set to the RMS of potential energy smaller than 0.001 Kcal Å^−1^ mol^−1^. For those flexible components, the most stable conformations were chosen from standard conformational analysis.

The X-ray crystal structures of 31 known targets and the putative targets related to CKD (Table 1) were downloaded from Protein Data Bank. The aforementioned structures were preprocessed. Hydrogen was added to the model, and its orientation was optimized using the CHARMm force field energy minimization while all non-hydrogen atoms were not allowed to move. The ligand position in target proteins was used to define the active site cavity. Docking protocol was performed to show the interaction with CKD target proteins and known target proteins using AutodockTools. This work was conducted using freely available software called AutoDock Vina [46].

The docking score between known drugs and molecular-protein complex, and known targets proteins was used as the cutoff value in this protocol (Table 1). For all target proteins, if the docking score of a natural product and a target was less than the corresponding cutoff value of positive drugs, and also less than −5.0 kcal/mol [47], they were considered to be effective docking and could be regarded as nodes. Their interaction could be further regarded as edges. As a result, natural product-target network could be constructed by cytoscape 2.8.

### Constructing Protein-protein Networks (PPIs) and Elucidating Biological Function Analysis

The construction of PPIs related to CKD was based on the protein expressed from a gene in a biological system. Although the mRNA expression level did not necessarily represent the true protein abundance, several studies have found mRNA and protein expression levels to be correlated [48], [49]. Therefore, we chose the proteins as nodes of the network corresponding to candidate genes obtained from the OMIM, GAD and GEO microarray data. The obtained candidate genes and known target proteins were mapped onto the human protein-protein databases including BIOGRID, INTACT, MINT, DIP, BIND and HPRD databases by a plugin named BisoGenet [50], and then PPI and known target protein-protein interaction network were constructed by Cytoscape 2.8. Extended protein-protein interaction network (EPPI) was further constructed by adding nearest neighbors of known drug target proteins. BisoGenet mapped intersection node between PPI and EPPI into human protein-protein databases in order to construct core protein-protein network (CPPI) of CKD.

Putative drug targets were subsequently screened out by using topological characteristic of CPPI, including degree [51], cluster coefficient [52], [53], betweeness centrality [54], bridgeness centrality [55], and closeness centrality [56]. The definitions of these measures are shown in Table 2. The protein nodes of CPPI were first sorted according to the value size of five topological parameters. The top 30% of nodes including more known protein targets were subsequently selected as putative drug targets. All topological algorithms are performed on large-scale computers by Matlab 7.0 program.

**Table 2:** Topological feature set.

Feature	Function	Description
Degree	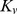	The number of links to node v.
Clustering Coefficient	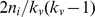	n_i_ is the number of links connecting the K_v_ neighbors of node v to each other.
Betweenness Centrality	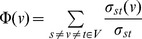	σ_st_ is the number of shortest paths between node s and t and σ_st_(v) is the number of shortest paths passing through a node v out of σ_st_
Bridging Centrality	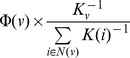	K(v) is the degree of node v, and N(v) is the set of neighbors of node v.
Closeness centrality	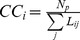	N_p_ is the total number of vertices in the graph and L_ij_ is the shortest path between vertices i and j.

The Kyoto Encyclopedia of Genes and Genomes (KEGG) pathway database was searched to elucidate the function of target proteins in the biological pathway. The statistical significance of each biological process was estimated by adopting both impact analysis and hypergeometric model (p≤0.01) algorithms of Pathway-Express (http://vortex.cs.wayne.edu/ontoexpress) [57].

### Constructing and Analyzing Three Types of Molecule-target Networks

In this work, the three molecule-target networks consisted of a natural product-target network, a main component-target network and a compound-target network. The natural product-target network was first constructed based on section ‘molecular docking and construction of natural product-target network’. The main component-target network was derived from the natural product-target network and was constructed by the interaction between main components from BSHX and their targets. Similarly, the compound-target network was constructed by the interaction between representative compounds from BSHX and their targets. These networks were analyzed based on the degree of topological parameters and literature survey.

### Experiments

Tanshinone IIA, rhein, curcumin, calycosin and quercetin (purity 99%) were purchased from the Chinese National Institute for the Control of Pharmaceutical and Biological Products (Beijing, China), Sixty-four Sprague-Dawley male rats (200±20 g) were obtained from animal center of Wenzhou Medical University. They were housed and cared for under a constant temperature (25±1°C) and humidity (50±10%) in animal antnun with grade SPF. Ethical approval for the study was obtained form Animal Care and Ethics Committee of Wenzhou Medical University. The rats were randomly divided into eight groups. These groups included sham group, Unilateral Ureteral Obstruction (UUO) group and UUO with Tanshinone IIA, Rhein, Curcumin, Calycosin, Quercetin and union therapy groups. All the UUO operations were performed using an established procedure [58]. Rats were successively administered with Tanshinone IIA (10 mg/kg/day), Rhein (10 mg/kg/day), Curcumin (25 mg/kg/day), Calycosin (10 mg/kg/day) and Quercetin (10 mg/kg/day) for 21 day after their operation. At the same time, sham operation group and model group were given the same volume of saline. On the 21^th^ day, serum from each of the different groups was collected. Blood urea nitrogen and creatinine in each serum were measured by a NOVA16 autoanalyzer (NOVA Biomedical, Waltham, MA).

## Results and Discussion

### Construction and Analysis of PPI Related to CKD

According to candidate genes obtained in Table S1, PPIs associated with CKD were constructed by the plugin BisoGenet. As shown in Figure. S1A, each node represented a protein and if two nodes linked each other, it was labeled an edge. There are 1008 nodes in Fig. 1A, including 486 isolated nodes and 522 nodes in 14 clusters. The biggest cluster consisted of 495 (49.1%) nodes, linked by 1155 (98.8%) edges.

In order to verify whether the constructed PPI accurately characterize the pathological processes associated with CKD, candidate genes were used for gene function analysis using Pathway-Express (*P*<0.01). The results were shown in Table S2 and indicated that the PPI are related to 36 signaling pathways, including a number of important and well known signaling pathways such as calcium signaling pathway [59], TGF-beta signaling pathway [60], [61], Jak-STAT signaling pathway [62], PPAR signaling pathway [63], [64], ECM-receptor interaction [65], renin-angiotensin system [66]–[68], VEGF signaling pathway [69], [70], focal adhesion [71], [72], insulin signaling pathway [73], [74] and MAPK signaling pathway [75], [76], primary immunodeficiency [77] and B cell receptor signaling pathway [78]. This suggests that CKD was involved in many signaling pathways and the PPI can accurately represent the complex pathogenesis of CKD. In addition, PPI was also related to many other signaling pathways that are still not reported, such as a neuroactive ligand-receptor interaction, adipocytokine signaling pathway, renal cell carcinoma and pancreatic cancer. These signaling pathways may provide some important clues for further research into signaling pathway of CKD.

### Constructing CPPI and Screening Out Putative Targets

**Figure 2:**
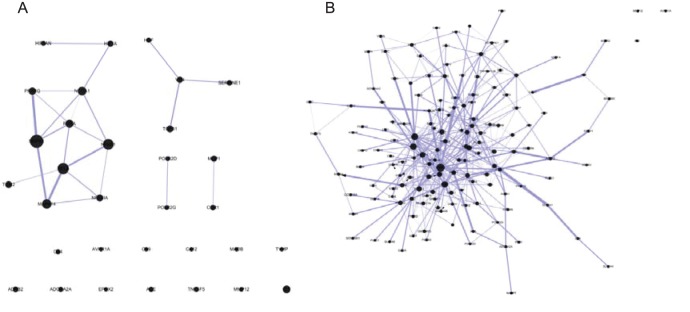
31 known target proteins network (A) and core protein-protein network (B). In this figure, each node is a protein, with two proteins being connected if there are interactions with each other. The size of each node is proportional to the degree number of proteins and the link thickness is proportional to the value of betweeness centrality among two connected proteins.

Making use of PPI was important for screening out new drug targets [79], [80]. The PPI of 31 known targets was shown in Fig. 2A. 31 known drug targets formed 4 clusters. The biggest cluster consisted of 11 nodes and was connected by 18 edges. Network characteristics indicated that the majority of drug targets tend to form an inner-interaction among each other. In addition, the network included 13 isolated nodes and 3 small clusters with 8 nodes. To further investigate if there was inner-interaction between the nodes in clusters and isolated nodes, we added the nearest neighbors of 31 known drug target proteins in order to obtain EPPI, as shown in Figure. S1B.

This network consisted of 1239 nodes, resulting in forming a huge cluster. Interestingly, by adding the nearest neighbors, 31 known target proteins can be connected to form a cluster. These phenomena further suggest that there may be a direct or indirect universal link among drug targets of CKD, which provide important clues and ideas for the discovery of new drug targets from the known drug targets. Prof. Li compared the topological properties of drug–targets with those of the non–drug-target sets, by mapping the drug targets in DrugBank to the human protein interaction network. Their results demonstrate that the drug-targets connect closely with each other and preferentially interact with other drug-targets [81]. Based on the above viewpoints, we considered that new drug targets of CKD have the largest probability in the EPPI.

The pathogenesis of CKD has not been clarified and the relevant literature and experimental data is still limited [82], [83]. However in this work, the constructed PPI not only can represent the primary pathological process of CKD (containing multiple known signal pathways), but also suggest the other potential signaling pathway, which provides academic clues for the study on the pathological mechanism of CKD. Therefore, we believe that the intersection nodes of PPI and EPPI can not only reflect the complex pathological process of CKD, but can also indicate that the nodes of intersection were most likely to become a new drug target. As shown in Fig. 2B, CPPI consisted of 156 nodes, and formed two clusters and 6 isolated nodes. One of the biggest cluster comprised a total of 148 (94.9%) nodes by linking 410 (99.8%) edges.

**Figure 3:**
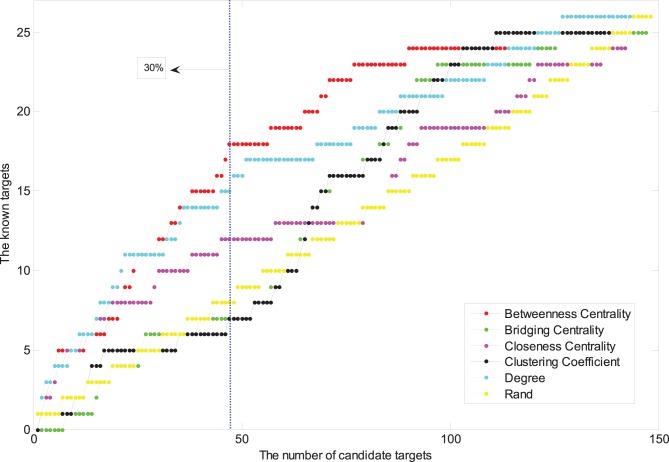
The predictive rate of different topological parameters.

In order to screen out the putative drug targets from CPPI, topological parameters including node degree, betweeness centrality, bridgeness centrality, closeness centrality and clustering coefficient, were used to predict the importance and accuracy of the protein nodes. We plotted the number of all protein nodes via the number of nodes of known drug target proteins, as shown in Fig. 3. The number of nodes of known drug target proteins increased with that of all nodes, but in the top 30% of nodes, the number of known drug target proteins increased more significantly based on the rank of betweeness centrality. 14, 18, 7, 6 and 6 known drug target proteins were included according to the rank of degree, betweeness centrality, bridgeness centrality, closeness centrality and clustering coefficient, respectively. In other words, betweeness centrality has a relatively higher predictive accuracy of known drug targets. The betweeness centrality of nodes can characterize a hub position in the network. A node with a large betweenness centrality but a small degree, if it was loss in a network, would result in the emergence of many modules. Therefore, the node with a larger betweenness centrality plays an important role in the network, and is key transmit point of biological information flow, and with a bigger probability of being a new drug target [84], [85]. Thus we selected the top 30% of nodes, which contained 31 protein nodes, as putative drug targets. As a result, except for the 25 target proteins without PDB X crystallization data or ligands, the surplus 37 target proteins were used to dock between the natural products and drug targets (Table 1).

### Construction and Analysis of Natural Product-target Network

As shown in Table S3, a total of 774 molecules were collected from Comprehensive Natural Products in TCM and Reaxys databases. These molecules included 458 compounds from AR, 118 compounds from RR, 74 compounds from CS, 108 compounds from SM and 48 compounds from CR. There are 32 molecules which coexist in two or more herbs.

The score of molecular docking was an important index to judge the binding degree between molecules and targets. In this research, the docking score between positive drugs and targets was shown in Table 1. If their docking scores were less than the cut-value and −5 kcal/mol, we believe that there is an interaction between the natural product and targets. Cytoscape, as shown in Figure. S2, subsequently constructed natural product-target network. This network consisted of 366 compounds and 37 drug targets that were linked by 1192 edges.

As shown in Figure. S2 and Table S4, 108 compounds from SM acted on average 6.1 targets. 118 compounds from RR acted on an average of 2.6 targets. 458 compounds from AR acted on an average of 2.3 targets. 74 compounds from CS acted on an average of 2.1 targets. 48 compounds from CR acted on an average of 1.9 targets. Compared to the other four herbs, the compounds from SM acted on the most drug targets, which may reflect its network characteristics as the main treatment herbs. The compounds from CS and CR acted on fewer targets, which may reflect their network characteristics as adjuvant therapy.

In the natural product-target network, each compound interacted with an average of 2.78 targets and the compounds acting on each target were from two or more herbs, which intuitively characterize “synergy” laws of TCM. Similarly, many drug targets were acted on by more than one compound. Tissue-plasminogen activator, hepatocyte growth factor, angiotensin converting enzyme, thymidine phosphorylase, adenosine A2α receptor, hypoxia-inducible factor 1α and proto-oncogene tyrosine-protein kinase were examples of highly connected drug targets, whose degree of nodes are 87, 79, 74, 76, 71, 66 and 60, respectively. The average number of compounds per candidate target was 33.1, indicating that many drug proteins related to CKD might share similar binding patterns with natural product molecules, which in turn might reflect the pharmacological multi-component, and multi-target facet of TCM.

Network data structures are amenable to many sophisticated forms of computational analysis which can visualize important, non-obvious properties of nodes and the relationships between them [86], [87]. The topological analysis of the networks may offer insights into the biologically relevant connectivity patterns, and pinpoint highly key compounds or targets. The node degree, as one of the most basic properties of a network, is often investigated. The highly connected nodes are referred to as hubs [88]. In natural product-target network, those nodes that have effect on the treatment of CKD had a higher degree. For example, the degree of tanshinone IIA was 9. Tanshinone IIA can inhibit effect on TGF beta 1-smads signal pathway in renal interstitial fibroblasts [89]. The node degree of rhein from RR was 11. Rhein can markedly ameliorate renal interstitial fibrotic lesions, with reduced alpha-smooth muscle actin expression, and attenuate the deposition of fibronectin (FN) [90]. The degree of curcumin from CR was 9. It could inhibit LPS-induced renal MCP-1 mRNA expression [91], block the profibrotic actions of TGF-beta on HK-2 cells through the down-regulation of the smad signaling pathway [92], reduce TGF-beta-induced increases in plasminogen activator inhibitor-1 (PAI-1), TGF-beta1, FN and collagen I (Col I) [92], and give substantial protection against oxidative damage [93]. The degree node of calycosin from AR was 6. Calycosin promotes angiogenesis via activation of MAPK with the involvement of ERK1/2 and ER [94]. The degree node of quercetin from CS was 4, which is from its antioxidant that protects renal function [95].

Surely, the compounds with higher degree would be key players in each herbs. This also indicates that the network-based analysis is capable of extracting the key ingredients from herbs. In addition, it is worth noting that some compounds, whose activities remained unknown, could be a key player of consideration in the future study of BSHX. Especially five candidate compounds, i.e., dihydroisotanshinone II, salviamone, monodemethoxycurcumin, pulmatin, isobavachalcone and liquiritigenin, might be novel leads for treatment of CKD and are worthy of further research.

In this work, a total of 62 targets have been annotated to have significant relationship with the pathological processes of CKD. All of these proteins might mediate each biological process at every stage along the CKD continuum. ACTN1, ADORA2A, APOA1, CLU, FN1, FYN, HGF, HNF4A, ITGAL, LCK, MAPK1, MAPK14, PIK3CA, PIK3R1, PLAT, PRKCB, PTPN1, RAF1, SERPINE1 and TGFB1 were involved in biological process of blood coagulation. Coagulation formation led to hemodynamic changes in the glomeruli and promoted the proliferation of mesangial cells. Mesangial cell proliferation can further increase the expression of endothelin, resulting in deposition and thrombus formation of intravascular fibrin. ACTN1, APOA1, CLU, FN1, FYN, HGF, LCK, MAPK1, MAPK14, PIK3CA, PIK3R1, PRKCB, PTPN1, RAF1, SERPINE1, TGFB1, TNFSF5 were involved in the biological process of platelet activation. In normal states, platelets play an important role on the hemostasis and coagulation process, which can maintain the integrity and normal functions of the vascular wall. However, after platelet activation, it may produce irreversible aggregation and finally form clots. Clearly, the compounds interacting with these receptors are associated with thrombosis and hyperlipidemia, and therefore possibly leading to inhibition of blood coagulation, activation of the fibrinolysis, inhibition of platelet aggregation and tackiness, decrease of plasma viscosity, and ultimately, cure of the thrombosis. CA12, CLU, FOS, IGHG1, JUN, MAPK1, MAPK14, MIF, NFKB1, NFKBIA, TGFB1, MCP1, CCR1, CCR5, FCGR2B, IGHG1, IL8, VTN, APOA1, TNFSF5 and PPARG were involved in the biological processes of immune response. It is generally considered that the early onset of CKD is closely related with the immune response. After the sustained damage to renal tissue, B cells and T cells were recruited and activated, which made a large number of B cells, T cells and macrophages infiltrate tissues, then enter the injured renal tissue, and finally secrete some cytokines such as ROS. It further damages renal tissue and leads to tissue fibrosis. Therefore, if the compounds can intervene in the immune process, they may inhibit tissue damage and delay the development of the disease process.

HIF1A, MCP1, PGF, PLAT, RAF1, STAT5B, TGFB1, CA9, HIF1A, VHL and TGFBR1 were involved in the biological process of hypoxia. Hypoxia can lead to tubular EMT or apoptosis, activate resident fibroblasts and impair peritubular capillaries, thereby creating a cycle of chronic hypoxia and progressive kidney failure. The prevention of the anoxic process can thus decrease the damage of kidney tissue by hypoxia. SERPINE1, STAT5B, HIF1A, TGM2, ADORA2A, CCL5, CCR1, CCR5, EPHX2, FOS, IL8, ITGAL, KIT, MCP1, MIF, NFKB1, TGFB1 and TNFSF5 were well related to reactive oxygen species and inflammation. Infection will stimulate macrophages contributing to the excessive secretion of inflammatory cells and ECM abnormal accumulation, ultimately leading to tissue damage and clinical symptoms. Therefore, the regulation of these proteins may inhibit the proliferation of vascular smooth muscle cells and further control the processes of CKD. ACE, EPHX2, SERPINE1, FOS, MIF and PPAR alpha are all concerned with hypertension. Through the modulation to these proteins, the CKD may achieve the antihypertensive curative effect.

35, 35 and 31 of 36 targets are targets of SM, AR and RR, respectively (Table 3). This suggests that SM, RR and AR participated in most of the biological process associated with CKD, such as infection, hypoxia, immunological stress, blood coagulation, platelet activation and matrix formation. RR had antiplatelet, anticoagulant and radical scavenging activities [96], [97], preventing the progression of CKD [27]. AM ameliorates renal fibrosis by modulating HGF and TGF-beta in rats with unilateral ureteral obstruction [98] and its total flavonoid fraction have antihypertensive effect in hypertensive rats [99]. Salvianolic acids not only acted as reactive oxygen species scavengers, but also inhibited inflammation and metalloproteinases expression from smooth muscle cells, and regulated immune function [32] 23 and 14 of 36 targets (Table 3) were targets of CS and CR, most of which were related to blood coagulation, platelet activation and response to hypoxia. CS was evaluated for its adjuvant potentials on the cellular and humoral immune responses [100] and also for hormone levels, androgen receptor mRNA and protein level in the kidney and testicle [101]. Curcumin blocks multiple sites of TGF-beta signaling cascade in renal cells [92]. The results showed that five herbs participated in the regulation of various biological processes of CKD. But compared to CS and CR, SM, RR and AR can regulate more biochemical processes of CKD (acting on more target), which may reflect the network regulation characteristics of compatibility law of TCM.

**Table 3:** The targets of five herbs.

SM′ targets	AR′ targets		RR′ targets		CS′ targets	CR′ targets	
Plasminogen activator, tissue	Plasminogen activator, tissue		Acyl-CoA dehy- drogenase family member 8, mitochondrial		Plasminogen activator, tissue	Plasminogen activator, tissue	
FYN oncogene related to SRC, FGR, YES	FYN oncogene related to SRC, FGR, YES		Adenosine A2α receptor		Hepatocyte growth factor	FYN oncogene related to SRC, FGR, YES	
Carbonic anhydrase II	Carbonic anhydrase II		Angiotensin converting enzyme		Hypoxia-inducible factor 1α	Raf kinase	
Protein tyrosine phosphatase	Protein tyrosine phosphatase		Carbonic anhydrase II		Peroxisome proliferator-activated receptor	Placeta growth factor	
Raf kinase	Raf kinase		Carbonic anhydrase XII		Angiotensin converting enzyme	Hepatocyte growth factor	
Placeta growth factor	Placeta growth factor		C-C chemokine receptor type 1		Monoamine oxidase B	Hypoxia-inducible factor 1α	
Macrophage migration inhibitory factor	Macrophage migration inhibitory factor		E3 ubiquitin protein ligase		Mitogen-activated protein kinase 1	Carbonic anhydrase XII	
Hepatocyte growth factor	Hepatocyte growth factor		Epidermal growth factor receptor		Hepatocyte nuclear factor 4-alpha/steroid receptor coactivator-1	Peroxisome proliferator-activated receptor	
Hypoxia-inducible factor 1α	Hypoxia-inducible factor 1α		FYN oncogene related to SRC, FGR, YES		Acyl-CoA dehydrogenase family member 8, mitochondrial	Hepatocyte nuclear factor 4-alpha/steroid receptor coactivator-1	
Carbonic anhydrase XII	Carbonic anhydrase XII		Glucocorticoid receptor		Mast stem cell growth factor receptor	Mast stem cell growth factor receptor	
Peroxisome proliferator-activated receptor	Peroxisome proliferator-activated receptor		Heat shock cognate 71 kDa protein		Small inducible cytokine A5	Thymidine phosphorylase	
Angiotensin converting enzyme	Angiotensin converting enzyme		Hepatocyte growth factor		Thymidine phosphorylase	Soluble epoxide hydrolase	
Monoamine oxidase B	Monoamine oxidase B		Hepatocyte nuclear factor 4-alpha/steroid receptor coactivator-1		Macrophage metalloelastase	Insulin receptor	
Mitogen-activated protein kinase 1	Mitogen-activated protein kinase 1		Hypoxia-inducible factor 1α		Mitogen-activated protein kinase 14	β2 adrenergic receptor	
Hepatocyte nuclear factor 4-alpha/steroid receptor coactivator-1	Hepatocyte nuclear factor 4-alpha/steroid receptor coactivator-1		Insulin receptor		Epidermal growth factor receptor		
Acyl-CoA dehydrogenase family member 8, mitochondrial	Acyl-CoA dehydrogenase family member 8, mitochondrial		Macrophage migration inhibitory factor		Insulin receptor		
Mast stem cell growth factor receptor	Mast stem cell growth factor receptor		Mast stem cell growth factor receptor		Proto-oncogene tyrosine-protein kinase LCK		
Small inducible cytokine A5	Small inducible cytokine A5		Mitogen-activated protein kinase 1		β2 adrenergic receptor		
Thymidine phosphorylase		Thymidine phosphorylase		Mitogen-activated protein kinase 14	Heat shock cognate 71 kDa protein		
Macrophage metalloelastase		Macrophage metalloelastase		Monoamine oxidase B	Phosphatidylinositol-4,5-bisphosphate 3-kinase catalytic subunit alpha isoform		
C-C chemokine receptor type 1		C-C chemokine receptor type 1		Phosphatidylinositol-4,5-bisphosphate 3-kinase catalytic subunit alpha isoform	Glucocorticoid receptor		
Vasopressin V1α receptor		Vasopressin V1α receptor		Placeta growth factor	Tyrosine-protein kinase BTK		
Soluble epoxide hydrolase		Soluble epoxide hydrolase		Plasminogen activator, tissue	E3 ubiquitin protein ligase		
Mitogen-activated protein kinase 14		Mitogen-activated protein kinase 14		Protein tyrosine phosphatase			
Epidermal growth factor receptor		Epidermal growth factor receptor		Proto-oncogene tyrosine-protein kinase LCK			
Insulin receptor		Insulin receptor		Raf kinase			
Protein kinase C, beta		Protein kinase C, beta		Small inducible cytokine A5			
Proto-oncogene tyrosine-protein kinase LCK		Proto-oncogene tyrosine-protein kinase LCK		Soluble epoxide hydrolase			
β2 adrenergic receptor		β2 adrenergic receptor		Thymidine phosphorylase			
Adenosine A2α receptor		Adenosine A2α receptor		Tyrosine-protein kinase BTK			
Heat shock cognate 71 kDa protein		Heat shock cognate 71 kDa protein		β2 adrenergic receptor			
Phosphatidylinositol-4,5-bisphosphate 3-kinase catalytic subunit alpha isoform		Phosphatidylinositol-4,5-bisphosphate 3-kinase catalytic subunit alpha isoform					
Glucocorticoid receptor		Glucocorticoid receptor					
Estrogen receptor		Tyrosine-protein kinase BTK					
E3 ubiquitin protein ligase		E3 ubiquitin protein ligase					

SM: *Salviae miltiorrhizae radix et rhizome;* AR: *Astragali radix;* RR: *Rhei radix et rhizome;* CS: *Cuscutae semen; CR: Curcumae rhizome.*

### Construction and Analysis of the Main Component-target Network

Although TCM contain several or even dozens of herbs and definitely include a variety of compounds, generally the number of therapeutic ones should be very limited because of low bioavailability, low content in the raw herbs, and so on [1], [102]. In other words, in most of the components it was difficult to attain a certain blood concentration to perform a therapeutic effect. We thus hypothesize that the effective components of TCM are the total absorbable bioactive compounds that reach certain concentrations in circulatory system [103], [104]. Therefore, in the natural product-target network, many components had little possibility to become effective substances.

Based on the ideas above, we selected a number of compounds with a larger amount of content and an appropriate bioavailability from Table S3 to construct a main component-network. As shown in Table S3, SM included water-soluble tanshinones and lipid soluble phenolic composition. According to their bioavailability, content and structure representation, danshensu, cryptotanshinone, tanshinone IIA and salvianolic acid B were selected as the main components from SM [31], [105], [106]. Similarly, chrysophanol, emodin, aloe-emodin, rhein, rhaponticin and gallic acid 3-O-β-D-Glucopyranoside from RR [107]–[109]; calycosin, formononetin, and calycosin glycoside calycosin-7-glucoside from AR [110]–[113]; hyperin, quercetin, kaempferol and β-sitosterol from CS [114]; curcumin, curcumol and curdione from CR [115]–[120] were selected as the main components, respectively.

**Figure 4:**
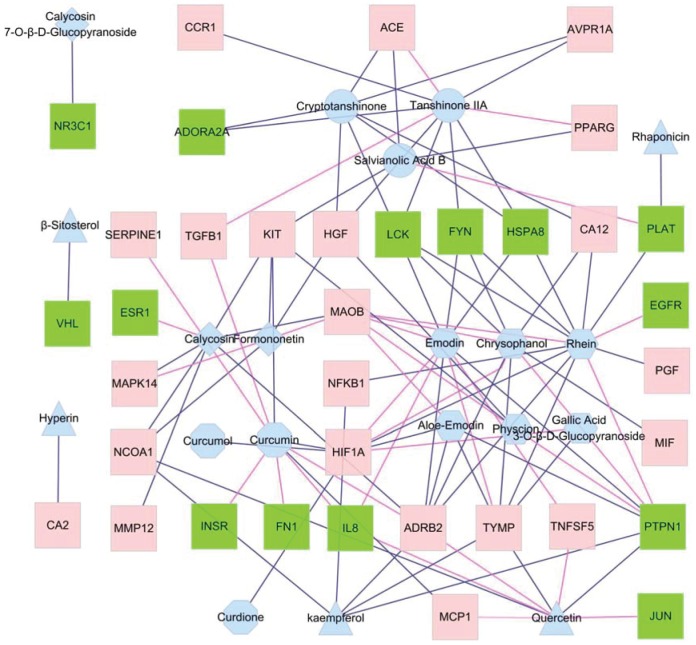
Main component-target network. Red and green square nodes represent the known and putative drug targets, respectively. Triangle, hexagonal, rhombus, octagonal and circle nodes represent main component from *Cuscutae semen*, *Rhei radix et rhizoma*, *Astragali radix*, *Curcumae rhizoma* and *Salviae miltiorrhizae radix et rhizoma*, respectively. If an edge color is purple-red, it means the interaction among two nodes had been confirmed by literatures. If an edge color is dark blue, it means the interaction among two nodes is validated by the calculation of molecular docking.

For a more comprehensive discussion of multi-component regulation on disease network, some of the 62 drug targets that have been reported in the literature, but were unable to be docked, were added in the network. The final constructed main component-network included 20 molecules and 36 drug targets, which were connected by 104 edges. As shown in Figure 4, it reflects the network relationship between the main components and their targets. The red and green dots represented the 22 known and 14 putative drug targets, respectively.

Table 4 listed the interaction between compounds and targets confirmed by previous literatures. As shown in Fig. 4, the literature-validated interaction between compounds and targets were connected with a red line. If the interaction of compounds and targets were not validated by literatures, they were connected with a dark blue line. As shown in Figure 4 and Table 4, it shows some interactions of main component-target can be documented. For example, emodin and monoamine oxidase B, hypoxia-inducible factor 1α, thymidine phosphorylase, macrophage metalloelastase, interleukin 8 and tumor necrosis factor ligand superfamily member 5 were linked directly in the network and their interaction relationship can be confirmed by literatures [121]–[126], suggesting the approach of network construction was the reliability. As shown in Fig. 4, emodin and proteins of PTPN1, HSPA8, LCK, ADRB2, and FYN were also connected directly, suggesting that emodin might interact with targets above. In addition, some literatures confirmed that some putative targets, such as estrogen receptor, FN 1, jun proto-oncogene, insulin receptor, tissue-plasminogen activator, interleukin 8 and epidermal growth factor receptor can interact with certain compounds from herbs, which indicated that these targets screened by CPPI could become a new drug target and further validate the reliability of approach.

**Table 4:** Literatures of interaction between compounds and targets.

Compounds	Targets
Emodin	Monoamine oxidase B [145], Hypoxia-inducible factor 1α [121], [158], Thymidine phosphorylase [136], Macrophage metalloelastase [123], Interleukin 8 [123], Tumor necrosis factor ligand superfamily member 5 [123]
Rhein	Monoamine oxidase B [145], Protein Tyrosine Phosphatase [141], Hypoxia-inducible factor 1α [137], Epidermal growth factor receptor [137]
Chrysorphanol	Monoamine oxidase B [145], Protein Tyrosine Phosphatase [141]
Aloe-emodin	Monoamine oxidase B [145]
Physcion	Monoamine oxidase B [145], Protein Tyrosine Phosphatase [141]
Gallic Acid 3-O-β-D- Gluco- pyranoside	Hypoxia-inducible factor 1α [159]
Salvianolic acid B	Tissue plasminogen activator [132], Plasminogen activator inhibitor-1 [132], Angiotensin converting enzyme [140]
Tanshinone IIA	Transforming growth factor beta 1 [134], [160], Angiotensin converting enzyme [134], Peroxisome proliferator-activated receptor [161], [162]
Calycosin	Estrogen receptor [94]
Formononetin	Monoamine oxidase B [146], Mitogen-activated protein kinase 14 [135]
Quercetin	β2 adrenergic receptor [163], Interleukin 8 [139],Tumor necrosis factor ligand superfamily member 5 [138], C-C motif chemokine-2 [138], Nuclear factor NF-κB [139], Jun proto-oncogene [138]
Curcumin	β2 adrenergic receptor [164], [165], Insulin receptor [92], C-C motif chemokine-2 [91], Interleukin 8 [91], Transforming growth factor beta 1 [92], Plasminogen activator inhibitor-1 [92], Fibronectin 1 [92]

In Figure 4, the compounds from RR, such as chrysophanol, emodin, physcion, aloe-emodin, rhein and gallic acid 3-O-β-D-Glucopyranoside can interact with drug targets, such as KIT, MAOB, ADRB2, EGFR, HIF1A, NFKB1, TNFSF5, PTPN1, IL8, EPHX2, TYMP, MIF, PLAT, LCK, PGF, HGF, FYN, CA12 and HSPA8. EGFR, TNFSF5, MIF and PGF were the unique targets of RR, and FYN, CA12, LCK and HSPA8 were common targets of RR and SM. HGF, PLAT and KIT were common targets of RR, SM and CR. PPARG, TGFB1, CA12, HSPA8, HGF, FYN, CCR1, LCK, ADORA2A, ACE, PLAT, AVPR1A and KIT directly linked with main components from SM, while ADORA2A, CCR1, ACE, PPARG and AVPR1A were the unique targets of SM. TGFB1 was the common targets of RR and CR. NR3C1, ADRB2, MMP12, MAPK14, MAOB, HGF, NCOA1, ESR1 and KIT interacted with the main components from AR, while MMP12, MAPK14 and ESR1 were the unique of AR. NCOA1 was the common targets of CR and AR. MAOB was the common targets of both AR and RR. ADRB2 was the common targets of RR, CR and AR. CA2, VHL, IL8, TNFSF5, PTPN1, TYMP, ADRB2, NFKB1, JUN and NCOA1 were directly connected with the main components from CS, and, CA2, JUN and VHL were the unique targets of CS. IL8, TNFSF5, PTPN1 and TYMP were the common targets of CS and RR. KIT, ADRB2, HIF1A, NFKB1, IL8, NCOA1, TGFB1, FN1, MCP1, SERPINE1, INSR and HGF were closely related to main components from CR, and, FN1, MCP1, INSR and SERPINE1 were the unique targets of CR. NFKB1, HIF1A and IL8 were common targets of CR, RR and CS. The relationship between the main components and target proteins indicated that the single herbs could not only interact with multiple targets, but also have their own unique targets. On the molecular level, it reflected both, the mutual coordination and the independence among multi-herbs, which indicated the overall network characteristics of complementary efficacy among different herbs.

Advances in pathophysiological research suggested that the CKD continuum begins with risk factors that initiate the process that leads to tissue damage. The pathophysiological continuum includes the hypoxia [127], inflammatory processes [128], blood coagulation [129], immune responses [130], and much more. Collectively, these risk factors might alter the expression of proteins in multiple cellular pathways, which lead to changes at the individual cell level, the tissue level and, ultimately, the disease state. The strategy behind the modern pharmaceuticals is to restore the healthy state by inhibiting a molecular target that is central to the mechanism of disease. However, a greater understanding of the CKD network reveals that the inhibition of an individual target is insufficient to restore the system to a healthy state. In these cases, modulating the activity of multiple targets would be required to achieve optimal therapeutic benefit [131]. TCM’s mechanism of action has postulated that the active compounds targeted at multiple proteins in the biological network and that the biological system would attain a new equilibrium in order to reduce a harmful impact.

The disorder of coagulation processes and fibrinolytic balance is an important pathophysiological change in the development process of glomerular sclerosis. Thrombin increases the fibrin deposition in glomeruli through upregulation of plasminogen activator inhibitor expression and inhibition of degradation of the mesangial matrix, which lead to accumulation of extracellular matrix and glomerulosclerosis. Sal B increased the fibrinolytic and anticoagulant potential by up-regulating the expression of tissue-type plasminogen activator and by down-regulating the expression of plasminogen activator inhibitor [132], which contribute to the balance of coagulation and fibrinolysis.

The injured renal tubular cell can secrete TGF beta cell factor, which can stimulate the secretion of FN, LN, fiber enzyme activator synthesis, inhibit matrix metalloproteinases (MMPs) expression and reduce the degradation of ECM, which all lead to the abnormal accumulation of extracellular matrix as the final result. Tanshinone IIA and curcumin significantly reduced the expression of angiotensin II, transforming growth factor beta, smad-3, collagen IV, plasminogen activator inhibitor-1, FN, collagen I mRNA and monocyte/macrophage either in the serum or kidney [92], [133], [134]. They inhibitd the profibrotic actions of TGF-beta through the down-regulation of the smad signaling pathway. Formononetin inhibits mitogen-induced proliferation, migration and extracellular matrix synthesis and down-regulates MAP kinase activity [135].

MCP-1 and IL-8 are two important pro-inflammatory cytokines and their upregulation will stimulate the proliferation of mesangial and epithelial cells, which lead to an increase of ECM synthesis of collagen and FN. Curcumin and quercetin significantly inhibited the expression and secretion of MCP-1 and IL-8 [62], [91]. Results displayed a decrease of the synthesis and accumulation of ECM. Therefore, AR, SM and CR can co-regulate TGF-beta/smad signaling pathway from different biological pathways and keep the accumulation and degradation of ECM in equilibrium.

In the case of hypoxia, hyperglycemia, proinflammatory cytokines and other stimuli, kidney cells can release a chemokine. With the promotion of chemokines, inflammatory cells infiltrated into tubulointerstitial fibrosis, produced fibrosis factor EGF-2, induced tubular cells into myofibroblasts and secreted extracellular matrix, which turned the lesion from the glomerular to tubulointerstitial area into an aggravating illness. Emodin significantly reduced the production of proinflammatory cytokines, such as tumor necrosis factor-alpha, IL-6 and IL-8, matrix metalloproteinase under hypoxia, and attenuated the expression of VEGF and hypoxia inducible factor 1 alpha [123], [129]. It also enhances thymidine phosphorylase mRNA [136]. Rhein can inhibit the expression of hypoxia-induced factor-1 alpha, epidermal growth factor, and the phosphorylation of inhibitor of NF-kappa B under normoxic or hypoxic conditions [137]. Quercetin or kaempferol have inhibited the NF-kappa B activation and the resultant up-regulation of the pro-inflammatory genes [138]. Quercetin also attenuated TNF alpha-mediated phosphorylation of extracellular signal related kinase, Jun NH_2_ terminal kinase and peroxisome proliferator activated receptor [139]. These compounds control the occurrence and development of CKD through the regulation of inflammatory cytokines.

The Renin-angiotensin system plays an important role in regulating the blood volume and systemic vascular resistance, which directly contributes to the development of renal tubular sclerosis. ACE is an important protein of the renin-angiotensin signal pathway. Salvianolic acid A can interact with angiotensin-converting enzyme (ACE) [140] and may reduce the arterial pressure, ventricular afterload and the blood volume.

The relationship between kidney and glucose metabolism is closely related. The normal function of the kidney is one of the keys to maintaining glucose metabolism. Patients with CKD usually develop insulin resistance. Under severe insulin resistance, insulin secretion is significantly reduced and results in serious hyperglycemia. Chrysophanol, physcion and emodin showed moderated bioactivity against human protein tyrosine phosphatase in vitro [141], and they strongly enhanced insulin sensitivity [142]. Quercetin prevented the TNF alpha-mediated phosphorylation of insulin receptor substrate 1, protein tyrosine phosphatase kappa B gene expression and the suppression of insulin stimulated glucose uptake [139]. These compounds can promote insulin secretion and accelerate the decomposition of glucose.

Cell reconstruction would take place when renal cells were damaged by external stimulation. However, cell reconstruction processes can enable the capillary permeability to extend and divulge protein urine, which results in the decrease of both vascular regeneration and reconstruction of the abnormal capillaries, and eventually renal unit atrophy. Calycosin acts as a selective estrogen receptor modulator to promote angiogenesis and reduces barriers to cells reconstruction [94]. Beta-adrenergic receptor was an important protein of calcium signaling pathway. Quercetin and curcumin regulate beta-adrenergic receptor, which lead to a reduction in angiotensin II, α1 adrenergic receptors vasoconstriction and tubular sodium reabsorption [92], [93], [143], [144].

Monoamine oxidase B was found in the serum and connective tissue and can promote the maturation of connective tissue. In the process of collagen formation, it takes part in the final stages of maturation bridge formation that makes the collagen and elastin bind. Formononetin, emodin, rhein, chrysorphanol, aloe-emodin and physcion can significantly inhibit monoamine oxidase B, and can also reduce the formation of collagen [145], [146].

The above results showed BSHX exerted its therapeutic effect by multi-component, multi-channel regulation on the abnormal accumulation of extracellular matrix, the release of inflammatory cytokine and the balance of coagulation and fibrinolytic. These processes are mutually crossed (Fig. 2B), and form a complex PPI network. BSHX restored the balance of biological network and control the occurrence, and development of CKD by acting on multiple nodes of protein-protein network.

### Construction and Analysis of Representative Compound-target Network

Modern biology research has confirmed that multiple genes were involved in complex diseases, multiple biochemical processes and multiple signaling pathways. When a drug is acting on a single drug targets, it is difficult to get the desired effect. Due to the robustness of biological networks, if certain biological information flow were to be blocked by a single drug, neighboring biological information flow can compensate for its loss and resist the influence of an external single factor. However, when attacked by a plurality of network nodes, the network is more susceptible. Therefore, the development of multi-component combination drugs has become an important strategy in the discovery of drugs for the treatment of complex disease. Chinese medicine was a clinical medicine experience summarized by ancient physicians, and it has developed a unique theory of Chinese medicine. According to TCM’s theory, the essence of TCM is the correct combination of a variety of natural products. After almost 3,000 years of continuous optimization in clinical practice, TCMs became better in terms of medication safety and possessed fewer side effects [147]. Therefore, TCM formula provides an important source of multi-target drug discovery for sophisticated diseases, and also contains a huge potential for development of multi-target drugs [127]–[128], [131]–[132].

**Figure 5:**
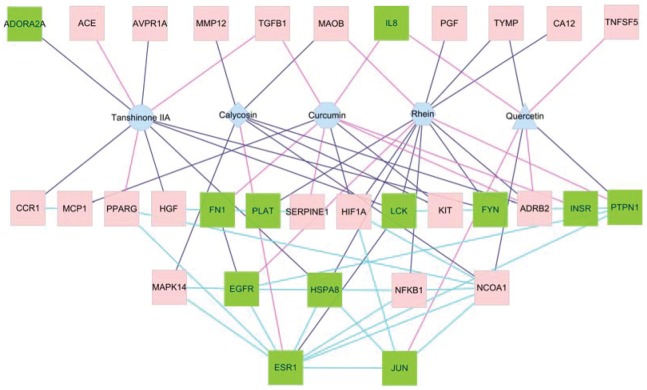
Compound-target network. Red and green square nodes represent the known and putative drug targets, respectively. Triangle, hexagonal, rhombus, octagonal and circle nodes represent main components from *Cuscutae semen*, *Rhei radix et rhizoma*, *Astragali radix*, *Curcumae rhizoma* and *Salviae miltiorrhizae radix et rhizoma*, respectively. If two nodes were connected by a purple-red line, it mean the interaction among two nodes had been confirmed by literatures. If two nodes were connected by a dark blue line, it mean the interaction among two nodes was validated by the calculation of molecular docking.

To explore combination drugs from BSHX, according to the network characteristics of nodes in Figure 4, we screened for the potential effective substance from each herb in BSHX. As shown in Figure 4, the node degree of tanshinone IIA from SM is 10, including CCR1, HGF, FYN, LCK, TGFB1, AVPR1A, PPARG, HSPA8, ACE and ADORA2A. These nodes were the main targets of SM and all unique targets, such as CCR1, AVPR1A, PPARG, ACE and ADORA2A. Therefore, tanshione IIA was selected as the representative compound. Similarly, the node degree of rhein from RR is 11, which included the main targets such as CA12, FYN, NFKB1, LCK, PTPN1, HSPA8, ADRB2, PLAT, HIF1A, TYMP and MAOB, and unique targets of EGFR and PGF. Therefore, rhein was selected as the representative compound. The node degree of calycosin from AR is 7, and includes main targets such as KIT, NCOA1, ADRB2 and MAOB, and all unique targets of ESR1, MAPK14 and MMP12. Therefore, calycosin was selected as representative compounds. The node degree of curcumin from CR is 9, which includes main targets, such as IL8, KIT, TGFB1, ADRB2, HIF1A, and all unique targets of MCP1, SERPINE1, INSR and FN1. Curcumin was thus taken as the representative compound. The node degree of quercetin from CS was 7, which includes most of targets, such as IL8, NCOA1, PTPN1, ADRB2, TNFSF5 and TYMP, and the unique targets of JUN. Quercetin was thus selected as the representative compound. According to the interaction of these five compounds and their targets, we constructed a representative compound-target network. At the same time, we integrated compound-target network and the known target protein-protein interaction network (Fig. 2A) into a compound-target-target network. As shown in Fig. 5, the five representative compounds can represent the synergism interaction among herbs, but also reflected the unique efficacy of each herb. These five compounds can therefore represent the peculiar, global and local effects and features of herbs, and were the most representative effective compounds of the whole herbs. Therefore, we believe that these five compounds can be redesigned as molecular combination drugs to treat CKD.

The pathogenesis of chronic kidney disease is closely related to blood coagulation. The biochemical process covers adenosine ADORA2A, TGFB1, HGF, FN1, PLAT, SERPINE1, LCK, FYN, PTPN1 and MAPK14 proteins shown in Fig. 5. As shown in the figure, tanshinone IIA and curcumin can jointly act on TGFB1 to downregulate the expression of TGFB and to reduce the deposit of extracellular matrix (ECM) [144], [148]. Quercetin and rhein can jointly act on PTPN1; tanshinone IIA and rhein can jointly act on FYN and LCK. These components inhibit blood coagulation and reduce thrombus formation by regulating different proteins in blood coagulation. TGFB1, TNFSF5, HGF, LCK, FYN, PTPN1 and MAPK14 are related to platelet activation. Fig. 5 shows that curcumin can act on FN1 and that tanshione IIA can act on HGF. FN1 and HGF can interact to jointly promote the degradation of ECM [92]. Similarly, quercetin and rhein can jointly act on PTPN1; tanshinone IIA and rhein can jointly act on FYN. Five components suppress platelet accumulation by inhibiting different proteins involved in platelet activation. Immune response is regarded as the source of chronic kidney disease. TGFB1, CA12, MCP1, PPARG, MAPK14, NFKB1 and JUN are closely related to immune response. Fig. 5 shows that curcumin and quercetin can jointly act on IL8 and JUN [149]; curcumin can act on MCP1, which can interact with CCR1 acted on by tanshione IIA. Calycosin can act on MAPK14, which interacts with EGFR acted on by rhein. These components can maintain the normal immune process by acting on different proteins in immune response to reduce the secretion of ROS cytokine. ADORA2A, TGFB1, IL8, TNFSF5, CCR1, MCP1 and SERPINE1 are related to oxidation and inflammation. Curcumin and quercetin jointly act on IL8; curcumin and calycosin jointly act on KIT; and tanshione IIA and curcumin can act on CCR1 and MCP1 protein, respectively. CCR1 interacts with MCP1 [150], [151]. CCR1, MCP1, and IL8 are the main inflammation promoting factors. Four molecules can inhibit inflammation and protect cells against oxidation attacks by regulating the expression of these proteins. TGFB1, PGF, MCP1, PLAT and HIF1A are closely related to hypoxia. Curcumin can attenuate the expression of HIF1A and MCP1 [91], [152]; rhein can regulate the expression of PLAT. Tanshion IIA, curcumin, and rhein can regulate the expression of these proteins to improve the damage of hypoxia to the kidney tissue. Tanshion IIA and curcumin can act on ACE and SERPINE1, which are related to hypertension [153]. These molecules can reduce the arterial pressure by jointly regulating hypertension. The five components can elicit synergistic therapeutic effects by acting on different targets in blood coagulation, hypertension, hypoxia, immune response, and other biochemical processes.

**Figure 6:**
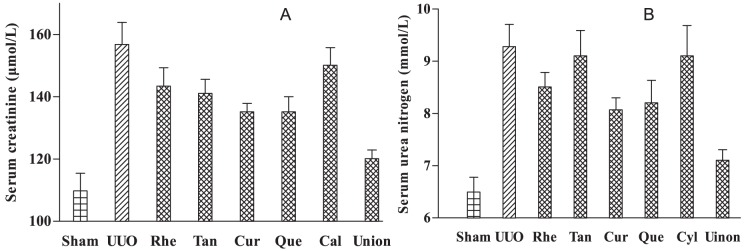
Levels of blood creatinine (A) and urea nitrogen (B) in different experimental groups including Rhein (Rhe), Tanshinone IIA (Tan), Curcumin (Cur), Quercetin (Que), Calycosin (Cal) and Union (five compounds).

We clarified whether or not the combined administration of these compounds has better effects than single administration. We selected important biochemical indicators (creatinine and urea nitrogen) for clinically diagnosing chronic kidney disease as the reference. We also determined differences in therapeutic effect between different component administrations. Renal fibrosis is a common way to develop various chronic kidney diseases into middle and late stages. We selected the UUO model to build a renal fibrosis model. Fig. 6A shows the creatinine contents in the sham operation group, model group, and model administration group after 21 days of treatment. Aside from calycosin, tanshinone IIA, rhein, curcumin and quercetin can reduce creatinine content (*p*<0.05). This result indicates that these compounds have therapeutic effects. Similar findings were reported by previous studies [90], [150]–[151], [154]–[155]. Creatinine content reduced more significantly after combined administration than after single administration of the four components. Significant differences in creatinine content were found between the model group and single administration of these components (*p*<0.01). Fig. 6B shows the contents of urea nitrogen for the sham operation group, model group, and model administration group. Aside from tanshinone IIA and calycosin, rhein, curcumin, and quercetin can significantly reduce the content of urea nitrogen (*p*<0.05). This result indicates that these compounds elicit therapeutic effects. Similar findings were reported by previous studies [150], [156]. Significant differences in the therapeutic effect of the three components were noted between the single and combined administration in the model group (*p*<0.01). Comparison of the contents of creatinine and urea nitrogen and the related literature show that the combined administration of the five compounds can significantly reduce these contents. The therapeutic effect of the combined administration is significantly higher than that of the single administration of the five compounds. These results prove the multi-component and synergistic mechanisms in TCM.

## Conclusion

Unlike the western medicine “one gene, one drug, one disease” research paradigm, TCM is seen as a unique system of theory, diagnosis and treatment tools in the world. Therefore, the research approach should be different from that of western medicine. In this work, an integral approach of network biology, chemoinformatics and bioinformatics was put forward to study effective substances and pharmacological mechanisms of TCM, which can better understand TCM’s mechanism of synergistic effects from more comprehensive viewpoints. The results indicated this approach could explain better network characteristics of the compatibility, and synergism principle of TCM, indicating that BSHX is regulated on CPPI by multi-component and multi-channel means. Our main findings are: 1) A novel approach is used to investigate the mechanisms of action of BSHX; 2) The developed different networks can be effectively applied to interpret the essence of ‘synergy’ and ‘compatibility’. It provides a new way to hold the inter-relationship between complex diseases and drug interventions through the network target paradigm for TCM [157]; 3) The constructed network system can pinpoint main active components and their corresponding targets, which will be helpful for therapeutic applications of TCM.

## Supporting Information

Figure S1 **Protein-Protein interaction networks associated with CKD.**
(EPS)

Figure S2 **Natural product-target network.**
(EPS)

Table S1 All selected genes from OMIM, GAD and GEO.
(XLSX)

Table S2 CKD associated with signaling pathways.
(XLSX)

Table S3 774 molecules collected from Comprehensive Natural Products in TCM and Reaxys databases.
(XLSX)

Table S4 The degree information of natural product-target network.
(XLSX)
